# Lead Integrity Alert Triggered by T-wave Oversensing

**DOI:** 10.19102/icrm.2020.110204

**Published:** 2020-02-15

**Authors:** Asad A. Aboud, Walter K. Clair

**Affiliations:** ^1^Division of Cardiovascular Medicine, Vanderbilt University Medical Center, Nashville, TN, USA

**Keywords:** T-wave oversensing, lead integrity alert, implantable cardioverter-defibrillator

## Abstract

T-wave oversensing (TWOS) is a relatively common occurrence in pacemakers and defibrillators that can lead to pauses and inappropriate implantable cardioverter-defibrillator shocks. We present a case of TWOS that triggered the Lead Integrity Alert (Medtronic, Minneapolis, MN, USA) without evidence of actual lead failure.

## Case presentation

**Figure 1:**
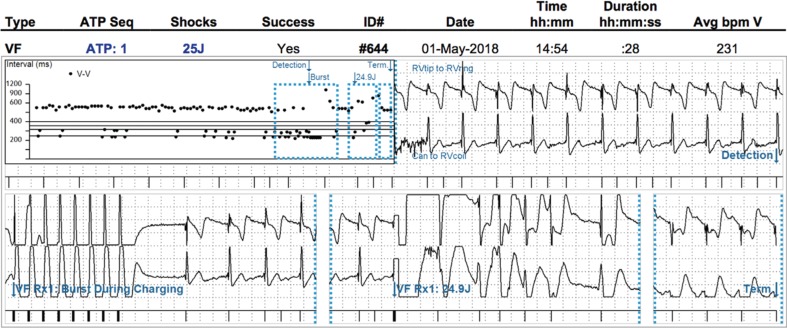
Inappropriate ICD therapy due to TWOS.

A 76-year-old male with a history of coronary artery disease and ischemic cardiomyopathy received a single-chamber Secura VR (Medtronic, Minneapolis, MN, USA) implantable cardioverter-defibrillator (ICD) in 2010 for primary prevention. A model 6935 lead (Medtronic, Minneapolis, MN, USA) was placed with the right ventricular (RV) lead tip in the RV apex, and the initial R-wave was recorded as 5.5 mV in a bipolar configuration. Eight years later, device interrogation revealed several episodes of nonsustained VT (NSVT) that, by electrogram analysis, were attributed to T-wave oversensing (TWOS). It was determined at that time that the TWOS was due to a low-sensed R-wave amplitude of 1.3 mV. A gradual decline in the R-wave amplitude was observed over the previous two years without a clear point of abrupt dropoff and without evidence of lead-position change on chest radiographs or lead impedance changes. No programming changes were made to the device at the time of TWOS diagnosis, and defibrillation threshold (DFT) testing was not attempted. Three months later, the patient received his first-ever ICD shock, and it was determined to be because of the TWOS **(Figure 1)**. As a result, the number of intervals for detection was changed from 18/24 for the ventricular fibrillation (VF) zone to 30/40 and from 16 beats for the ventricular tachycardia (VT) zone to 32 beats. The device continued to document NSVT due to TWOS in the month following the ICD shock, but no therapies were delivered following the aforementioned device adjustments.

**Figure 2:**
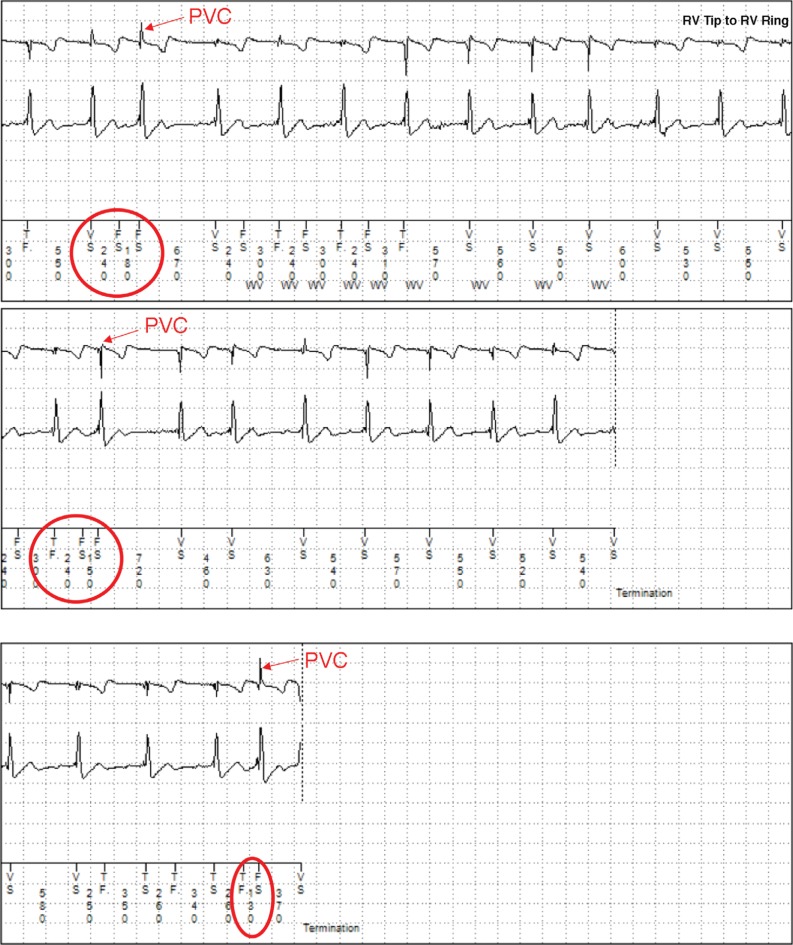
**A:** A LIA was triggered by NSVT with an average R–R interval of less than 220 ms (WV indicates that VT detection was overruled by the wavelet discrimination function). **B:** Frequent short V–V intervals (< 140 ms) attributed to PVCs following TWOS.

Two months later, a lead integrity alert (LIA) was received from the patient’s ICD based on frequent short V–V intervals (VVIs) on the sensing integrity counter and frequent NSVT episodes with an average cycle length of less than 220 ms **(Figure 2)**. However, there were no significant changes to the lead impedance or capture threshold observed. Electrogram analysis of the short V–V intervals revealed that premature ventricular contractions (PVCs) following TWOS were being interpreted by the device as short (< 140-ms) V–V-coupling intervals **(Figure 2)**. Based on this information, the RV lead-sensing configuration was changed from true bipolar to integrated bipolar, which resulted in a measured R-wave of more than 6 mV and eliminated the TWOS. Device interrogations performed over the following nine months revealed no subsequent occurrences of TWOS or LIAs.

## Discussion

TWOS after ICD implantation can lead to several issues including pauses, inappropriate ICD therapy delivery, and an inhibition of cardiac resynchronization.^1,2^ In this particular case, the combination of TWOS and PVCs was sufficient enough to cause short V–V coupling intervals, which resulted in inappropriate ICD therapy delivery as well as a false-positive LIA. To our knowledge, there has only been one previously described case of TWOS-triggered LIA.^3^ However, the management approach in the previously published case is different from the one we describe here.

Medtronic’s LIA (Minneapolis, MN, USA) protocol is a system designed to detect early signs of lead fracture and decrease the chances of receiving inappropriate therapies due to lead fracture by changing the programmed settings.^4–6^ LIA is triggered when at least two of the following three criteria are met: abnormal RV lead impedance (defined as an impedance that is significantly higher or lower than a calculated baseline impedance level), two or more NSVTs with intervals that are shorter than 220 ms, and at least 30 short VVI counts (< 140 ms) within three consecutive days.^7^ When LIA is triggered, the device automatically increases the number of intervals to detect VF to 30/40, which is intended to reduce the delivery of inappropriate shocks due to a lead fracture.

There are several ways by which to address TWOS, ranging from device reprogramming to lead revision,^8^ and each approach has different advantages and disadvantages. The TWOS in the previously reported case was successfully resolved by changing the sensitivity of the RV lead to enhance the discrimination between the detected R- and T-waves. However, such an approach would not have been effective in the case we report here, as the R-wave amplitude in the programmed configuration was extremely small relative to the amplitude of the T-wave. Fortunately, changing the RV lead’s sensing configuration from true (tip to ring) to integrated (tip to coil) bipolar is a programmable option among Medtronic devices that offers a noninvasive method by which to address this issue. Changing the RV lead-sensing configuration effectively addressed TWOS in this case without any evidence of far-field oversensing (a known disadvantage of this approach).^9^

Repeat DFT testing was not done after changing the sensing configuration, as the R-wave amplitude was measured as more than 6 mV in the integrated bipolar configuration. Moreover, unlike the previously reported case of TWOS triggering an LIA,^3^ the sensing threshold was not decreased in our case. Therefore, a compromise in the ability to detect VF was deemed extremely unlikely. That being said, when the ICD generator reaches its elective replacement interval, a shared decision-making appointment should be scheduled with the patient to discuss options should the lead be found to be compromised when invasively evaluated. Possible options for treatment could include the addition of a sensing-only lead, the addition of a new defibrillation lead, or the extraction of the model 6935 lead (Medtronic, Minneapolis, MN, USA) and implantation of a new defibrillation lead instead.
